# Optic-flow selective cortical sensory regions associated with self-reported states of vection

**DOI:** 10.3389/fpsyg.2015.00775

**Published:** 2015-06-08

**Authors:** Maiko Uesaki, Hiroshi Ashida

**Affiliations:** ^1^Department of Psychology, Graduate School of Letters, Kyoto University, KyotoJapan; ^2^Japan Society for the Promotion of Science, TokyoJapan

**Keywords:** vection, optic flow, self-motion, fMRI, multisensory, visual, vestibular, sensory integration

## Abstract

Optic flow is one of the most important visual cues to the estimation of self-motion. It has repeatedly been demonstrated that a cortical network including visual, multisensory, and vestibular areas is implicated in processing optic flow; namely, visual areas middle temporal cortex (MT+), V6; multisensory areas ventral intra-parietal area (VIP), cingulate sulcus visual area, precuneus motion area (PcM); and vestibular areas parieto-insular vestibular cortex (PIVC) and putative area 2v (p2v). However, few studies have investigated the roles of and interaction between the optic-flow selective sensory areas within the context of self-motion perception. When visual information (i.e., optic flow) is the sole cue to computing self-motion parameters, the discrepancy amongst the sensory signals may induce an illusion of self-motion referred to as ‘vection.’ This study aimed to identify optic-flow selective sensory areas that are involved in the processing of visual cues to self-motion, by introducing vection as an index and assessing activation in which of those areas reflect vection, using functional magnetic resonance imaging. The results showed that activity in visual areas MT+ and V6, multisensory area VIP and vestibular area PIVC was significantly greater while participants were experiencing vection, as compared to when they were experiencing no vection, which may indicate that activation in MT+, V6, VIP, and PIVC reflects vection. The results also place VIP in a good position to integrate visual cues related to self-motion and vestibular information.

## Introduction

When moving through any given environment, being able to accurately estimate one’s position, orientation, and displacement is crucial to successful navigation as well as safety. Coherent perception of self-motion is constructed by integrating sensory information, including visual and vestibular signals (Wertheim, 1994; Wexler et al., 2001).

Optic flow is the pattern of apparent motion on the retina caused by the relative motion between an observer and the scene, and is one of the most important visual signals for the estimation of self-motion (Gibson, 1950, 1954; Warren and Hannon, 1988).

It has been shown that there is a network of cortical sensory regions that respond selectively to optic flow (Duffy and Wurtz, 1991a,b, 1995; Cardin and Smith, 2010, 2011). Those regions are; visual areas middle temporal cortex (MT+), V6; multisensory areas ventral intra-parietal area (VIP), cingulate sulcus visual area (CSv), precuneus motion area (PcM); and vestibular areas putative area 2v (p2v) and parieto-insular vestibular cortex (PIVC).

Although generally, perception of self-motion depends on multisensory integration (Howard and Templeton, 1966), there are circumstances under which available sensory information is restricted. When visual information (i.e., optic flow) is the sole cue to computing self-motion parameters, the discrepancy amongst the sensory signals may arise and as a result, induce an illusion of self-motion (Dichgans and Brandt, 1978). This visually induced sensation of self-motion is referred to as ‘vection’ (Koenderink, 1986; Lappe et al., 1999). By introducing vection as an index, it might be possible to identify cortical sensory areas that are involved in integration of visual information related to self-motion and vestibular information (or lack thereof).

Kovacs et al. (2008) investigated cortical regions, of which activation is correlated with vection. In their study, participants passively viewed two types of optic-flow stimulus: One was perceived predominantly as a cue to self-motion and the other perceived predominantly as a cue to object-motion. By contrasting the cortical activation patterns during participants’ observation of the two types of optic-flow stimulus, they found that MT+, precuneus, an area corresponding to VIP in the right hemisphere, and areas corresponding to V6 and CSv in the left hemisphere. These findings are partially corroborated by Wall and Smith (2008) that suggests that areas MST, MT, CSv, and VIP exhibit differential responses to optic-flow stimuli that are compatible with self-motion.

A vestibular area corresponding to PIVC has also been implicated in cortical representation of vection: Brandt et al. (1998), Kleinschmidt et al. (2002), and Deutschlander et al. (2004) reported deactivation in PIVC while participants were experiencing vection.

Many of the cortical sensory areas that respond selectively to optic flow (Cardin and Smith, 2010, 2011) have been associated with vection. However, few studies have investigated the roles of and interaction between the optic-flow selective sensory areas within the context of self-motion perception, by functionally identifying those areas prior to the experiment, and directly correlating activation in those areas and presence of vection using a regions of interest (ROI) analysis. Consequently, the cortical sensory areas that are involved in integration of visual information related to self-motion and vestibular information are yet to be identified.

This study aimed to determine which of the optic-flow selective sensory areas are involved in the processing of visual cues to self-motion, by assessing whether optic flow is encoded differently according to the presence or absence of vection, and if so, activation in which of those areas reflect vection, using functional magnetic resonance imaging (fMRI).

## Materials and Methods

### Participants

Three healthy volunteers (two males and one female; of the ages between 25 and 47 years) participated in the study. All had normal or corrected-to-normal vision and were screened according to standard MRI exclusion criteria. Participants gave written informed consent to take part in this study, which was conducted in accordance with the ethical standards stated in the Declaration of Helsinki and approved by the local ethics and safety committees at Kyoto University.

### Data Acquisition

Magnetic resonance images were obtained with a 3-Tesla Siemens Magnetom Verio scanner (Siemens, Erlangen, Germany) and a Siemens 32-channel posterior-head array coil that gives improved signal-to-noise ratio in the occipital cortex at the expense of the anterior part of the brain. For each participant, a high-resolution T1-weighted 3D anatomical image was acquired [magnetization-prepared rapid-acquisition gradient echo (MP-RAGE), Siemens; 208 axial slices, 1-mm isotropic voxels, time of repetition (TR) = 2250 ms, time echo (TE) = 3.51 ms, field of view (FoV) = 256 mm × 256 mm, flip angle = 9°, bandwidth = 230 Hz/pixel]. This anatomical image was used as a reference to which the functional images were coregistered. The functional data were acquired with a gradient echo, echo-planner sequence (39 contiguous axial slices, 3-mm isotropic voxels, TR = 2000 ms, TE = 25 ms, FoV = 192 mm × 192 mm, flip angle = 80°, bandwidth = 2368 Hz/pixel). Each experimental run lasted 4 min 16 s for both the main experiment and the localizer. For coregistration purposes (see Data Analysis), between the functional and anatomical scans, a single-volume echo-planner imaging (EPI) sequence was acquired with the same position parameters as in the experimental runs.

### Stimuli and Procedure

Stimuli for both the main experiment and the localizer were displayed onto an in-bore rear-projection screen in the MRI machine by means of a liquid crystal display (LCD) projector, viewed via MaxTV binocular magnifier goggles (Eschenbach Optik GmbH, Nuremberg, Germany; the metal parts were removed) in order to maximize the stimulated area of the visual field. The images subtended 30° × 30° visual angle and had a resolution of 768 × 768 pixels with a refresh rate of 60 Hz. The stimulus for the main experiment was a random dot kinematogram consisting of 200 moving white square dots of 10 × 10 pixels (subtending approximately 0.4° × 0.4° visual angle), on a dark background. The dots, which initially appeared at random locations, formed a circular patch of 30° diameter. Motion directions of the dots were arranged so that the dots cycled through a spiral space with time-varying trajectories away from and toward the center of the patch. The trajectories were defined by the equations proposed in Morrone et al. (2000):

(1) $$\begin{array}{c} dr/dt=r\nu \,\cos\phi \,\\ d\theta /dt=\nu \,\sin\phi \end{array}$$

or

(2) $$\begin{array}{c} dr/dt=\nu \,\cos\phi \,\\ d\theta /dt=\left(\nu \,\sin\phi \right)/r, \end{array}$$

where (*r*, θ) corresponds to the position in the polar coordinates (0 ≤ *r* ≤ 1), ϕ determines the spiral direction of flow and *v* is the dot speed. ϕ was linearly increased at the rate of 0.25π/s (i.e., 8 s/cycle). Expansion and contraction simulated forward and backward motion of the observer, respectively.

Although the average speed of the dots was maintained at 3.54 pixel/frame (~ = 8.3°/s) throughout the experiment, the speed gradient was manipulated to create two conditions: Gradient condition and no-gradient condition. In the gradient condition [Eq. (1)], the speed of the dots increased linearly with eccentricity in all directions from the center (minimum: 0.625 pixels/frame ~ = 1.5°/s; maximum: 5 pixels/frame ~ = 11.7°/s). Dots near the center of the display moved slower whilst dots in the periphery moved faster, which made the pattern consistent with optic flow on the retina during self-motion in terms of speed. In the no gradient condition [Eq. (2)], no speed gradient was applied to the motion of the dots, i.e., the speed at which the dots moved was constant at all locations in the display, which is inconsistent with optic flow during self-motion.

**FIGURE 1:**
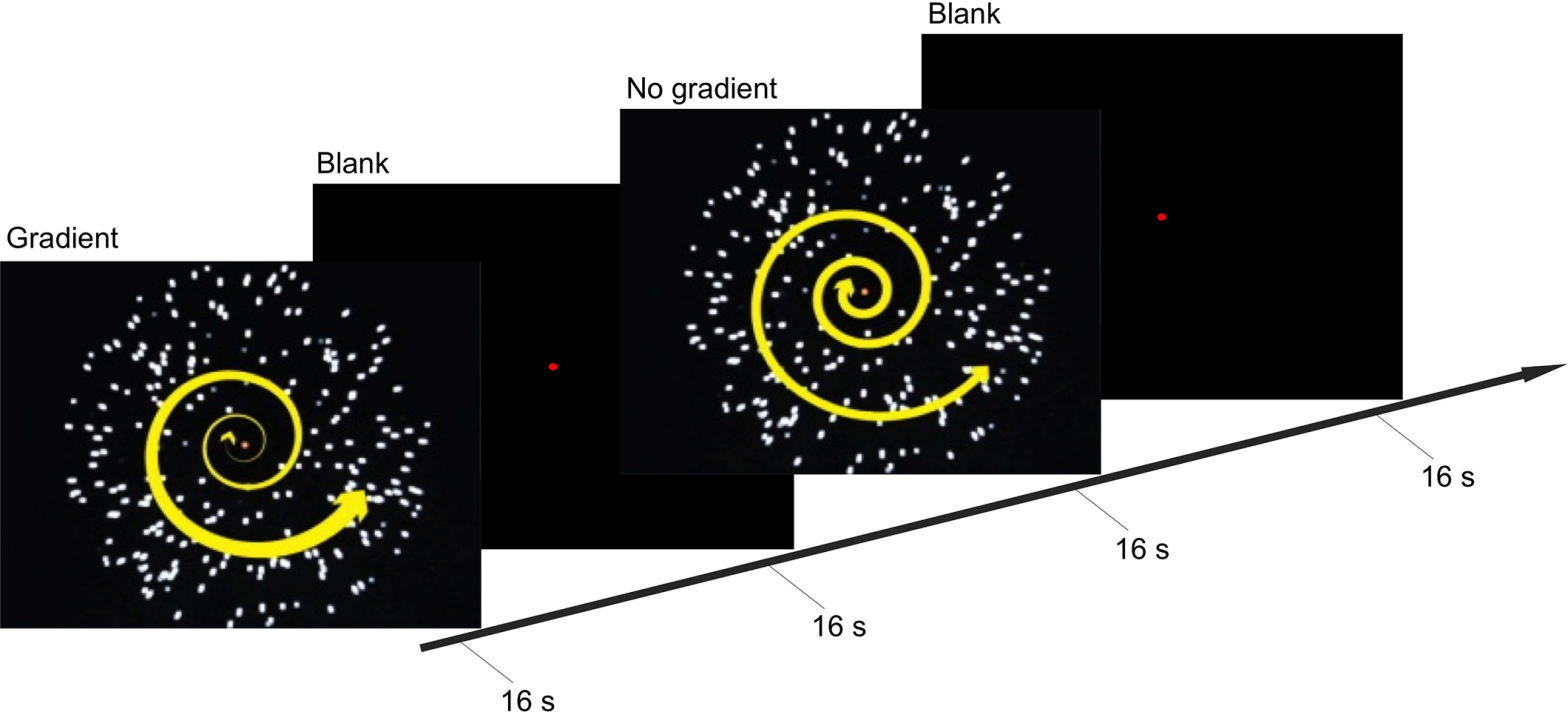
**Experimental procedure and stimuli**. Timeline illustrates the stimuli presented during the gradient and no-gradient conditions, and a blank screen with a fixation point presented during inter-stimulus interval (ISI). During the stimulus-presentation blocks, participants held down one of the two buttons to indicate whether they were experiencing vection or no vention.

These two conditions were presented in a block design in a back-to-back manner (i.e., gradient–no-gradient–no-gradient–gradient–gradient and so on; **Figure 1**). Within each block, one of the two types of optic-flow stimulus was presented for 16 s, followed by a 16-s inter-stimulus interval (ISI); and within each experimental run, there were eight stimulus blocks (four blocks per condition). Participants took part in 10 experimental runs (over two scans conducted on two non-consecutive days), each lasting 4 min 16 s.

The experiment also employed an event-related design to allow for a more direct inference on correlation between observed cortical activity and vection. During the stimulus-presentation phase of each block, participants pressed one of the two buttons to indicate whether or not they were experiencing vection. Pressing one of the two buttons indicated the onset of vection, the other indicated the onset of visual stimulation that was not accompanied by vection or the offset of vection. Consequently, a sequence of alternating button presses was recorded, identifying periods of time during which participants perceived themselves to be moving (vection) and to be stationary (no vection).

A red central fixation point was provided throughout the experimental runs, and participants were instructed to maintain fixation at all times.

### Functional Localizer

In order to quantify activity during the main experiment, various ROI previously associated with optic-flow processing or self-motion were identified with separate localizer scans, using the procedure based on that described in Pitzalis et al. (2010); in which a coherent optic-flow (similar to the stimulus presented in the gradient condition in the main experiment, but the direction of motion changed randomly every 500 ms) and a random-motion stimuli were presented in 16-s blocks.

The ROIs were defined as all contiguous voxels that were significantly more active with a pattern of expansion–contraction and rotation (a coherent optic-flow stimulus), than with a random-motion stimulus; in the middle temporal cortex (MT+), the parieto-occipital sulcus (V6), the VIP, the cingulate sulcus (CSv), the junction of the intraparietal sulcus (p2v), the region of the precuneus dorsal to the ascending arm of the cingulate sulcus motion area (PcM), and the posterior region of the insula (PIVC).

### Data Analysis

All data were preprocessed and analyzed with BrainVoyager QX (version 2.6, Brain Innovation, Maastricht, the Netherlands). EPIs were corrected for head motion and slice timing, and were filtered using a temporal high-pass filter with the cut-off of 3 cycles/run. No smoothing was applied. All functional images were aligned to the EPI acquired between the functional and anatomical scans. EPIs were first coregistered to the in-session MP-RAGE acquired with the 32-channel posterior-head coil. All images were then aligned to the reference MP-RAGE acquired with the full-head 32-channel phased-array coil, which were in alignment with the anterior–posterior commissure (AC–PC) axis. To obtain the coordinates of ROIs in a normalized anatomical space, all data were subsequently transformed to Talairach space. Duration of vection/no-vection events and the timing of these events were derived from participants’ button presses.

Analysis was conducted by fitting a general linear model (GLM). Head-motion parameters were not included as GLM regressors. Every stimulus block and every vection/no-vection event were convolved with a canonical haemodynamic response function. It was then entered into a multiple-regression analysis to generate parameter estimates for each regressor at each voxel. Effect size [i.e., percentage blood-oxygen-level-dependent (BOLD) signal change] for the two conditions and the two sets of events were extracted for each independently defined ROI by averaging across all voxels in the ROI.

## Results

### Perceived Vection

**FIGURE 2:**
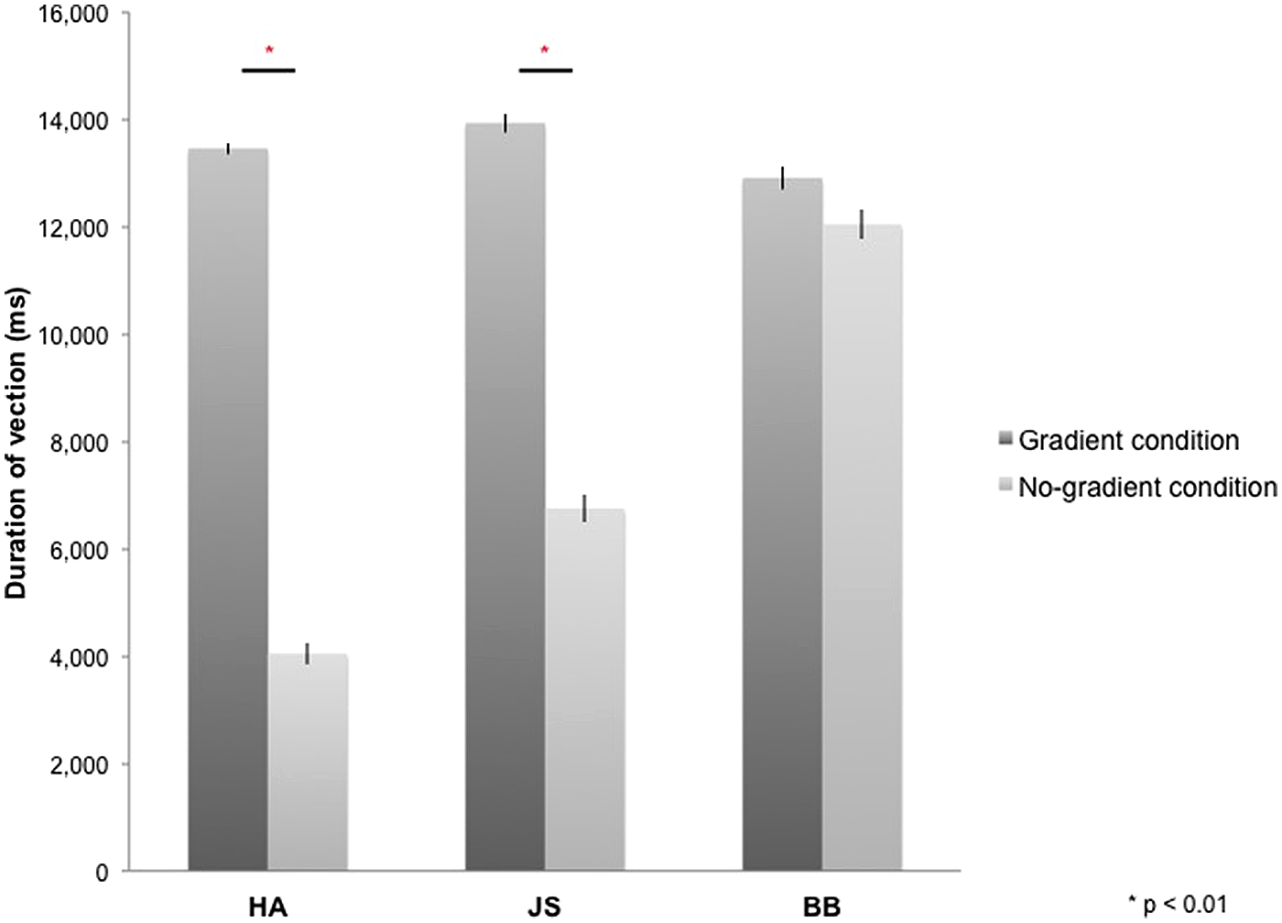
**Duration of vection perceived during 16-s stimulus blocks in gradient and no-gradient conditions averaged over 10 runs for each participant**. Error bars indicate SE.

All participants reported vection during optic-flow stimulation in the MRI scanner. The duration for which vection was perceived by each participant was averaged across four blocks of each experimental condition (i.e., gradient/no-gradient) and across 10 experimental runs (**Figure 2**). Vection was induced by both types of optic-flow stimulus with and without a speed gradient; however, two out of three participants reported more sustained perception of vection in the gradient condition [HA: *t*(9) = 21.59, *p* < 0.01; JS: *t*(9) = 8.97, *p* < 0.01; BB: *t*(9) = 2.07, *p* = 0.07; two-tailed].

### Functional Localization

To localize ROIs, BOLD responses to the coherent optic-flow and the random-motion stimuli were contrasted, which allowed for isolation of cortical sensory regions that are significantly more sensitive to coherent optic flow at *p*-value (uncorrected) of less than 0.005. The analysis was performed on the 3D anatomical volumes for each participant.

**FIGURE 3:**
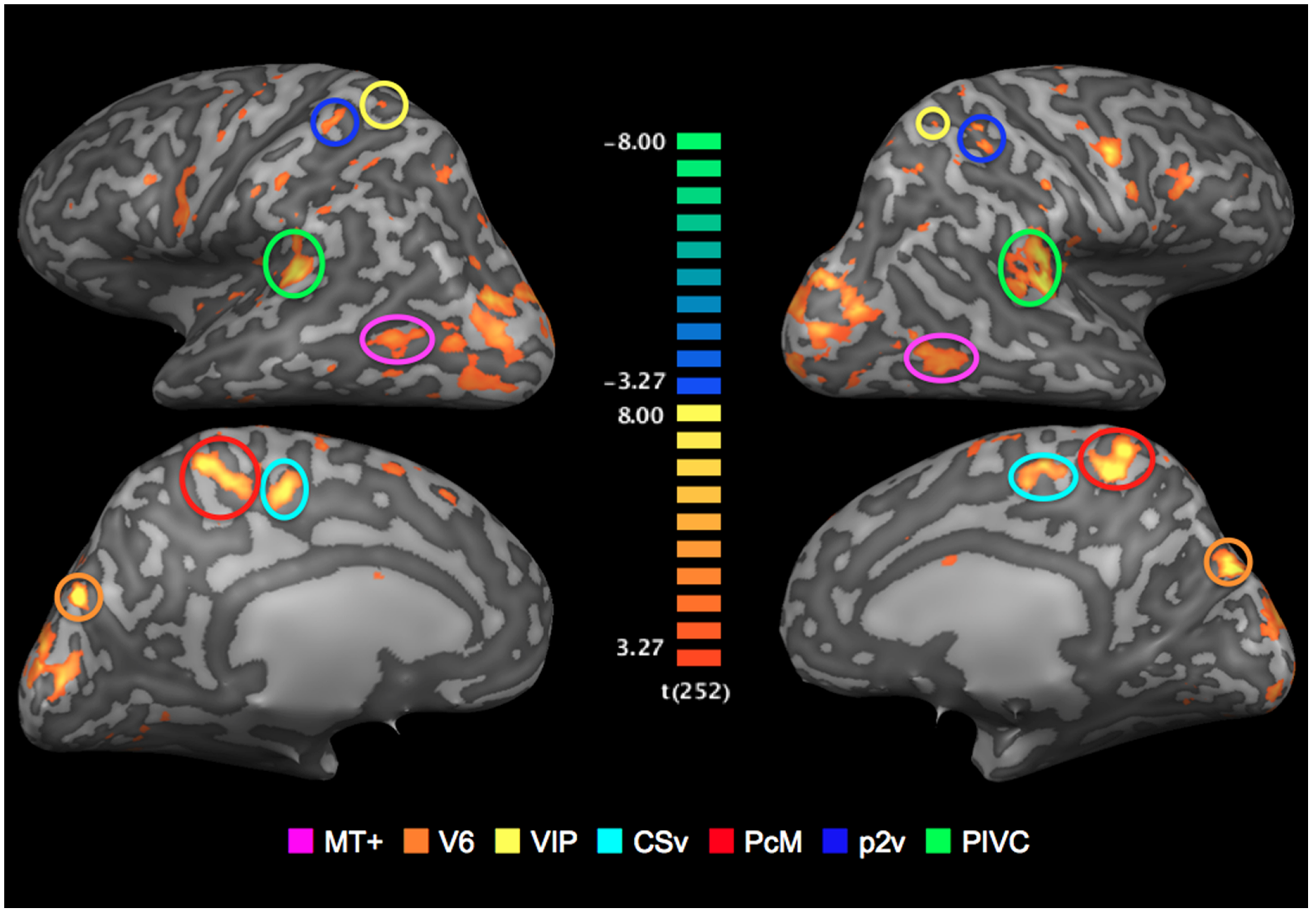
**Regions of interests (ROIs): Optic-flow selective sensory regions**. The map of areas that showed a significantly greater response to optic-flow stimulus than to random-motion stimulus is superimposed onto inflated representations of the left and right hemispheres of one representative brain. *T*-values are color-coded (see color bar). All activation shown is thresholded at *p* < 0.005 uncorrected.

All seven ROIs were successfully identified bilaterally for all three participants (**Figure 3**). The locations of those regions coincide with the Talairach coordinates of the counterparts reported in Cardin and Smith (2010). Mean Talairach coordinates for these ROIs are reported in **Table 1**, along with cluster sizes.

**Table 1:** Mean Talairach coordinates and cluster sizes in voxels of regions of interests (ROIs).

Left	*x*	*y*	*z*	Cluster size	Right	*x*	*y*	*z*	Cluster size
MT+	−44	−62	1	368	MT+	45	−59	3	499
V6	−14	−81	22	284	V6	14	−80	27	467
Ventral intra-parietal area (VIP)	−23	−56	44	74	VIP	20	−58	45	37
Cingulate sulcus visual area (CSv)	−14	−27	42	150	CSv	9	−27	46	119
Precuneus motion area (PcM)	−14	−49	47	74	PcM	8	−47	47	139
p2v	−30	−43	53	44	p2v	28	−42	53	47
Parieto insular vestibular cortex (PIVC)	−50	−38	18	566	PIVC	49	−35	18	718

### Main Experiment

#### Effect of Speed Gradient

Speed gradients are one of the physical components of optic flow perceived during self-motion. To examine whether activity in the sensory areas of interest is modulated by this physical property, activation in each ROI was contrasted between the gradient and the no-gradient conditions.

**FIGURE 4:**
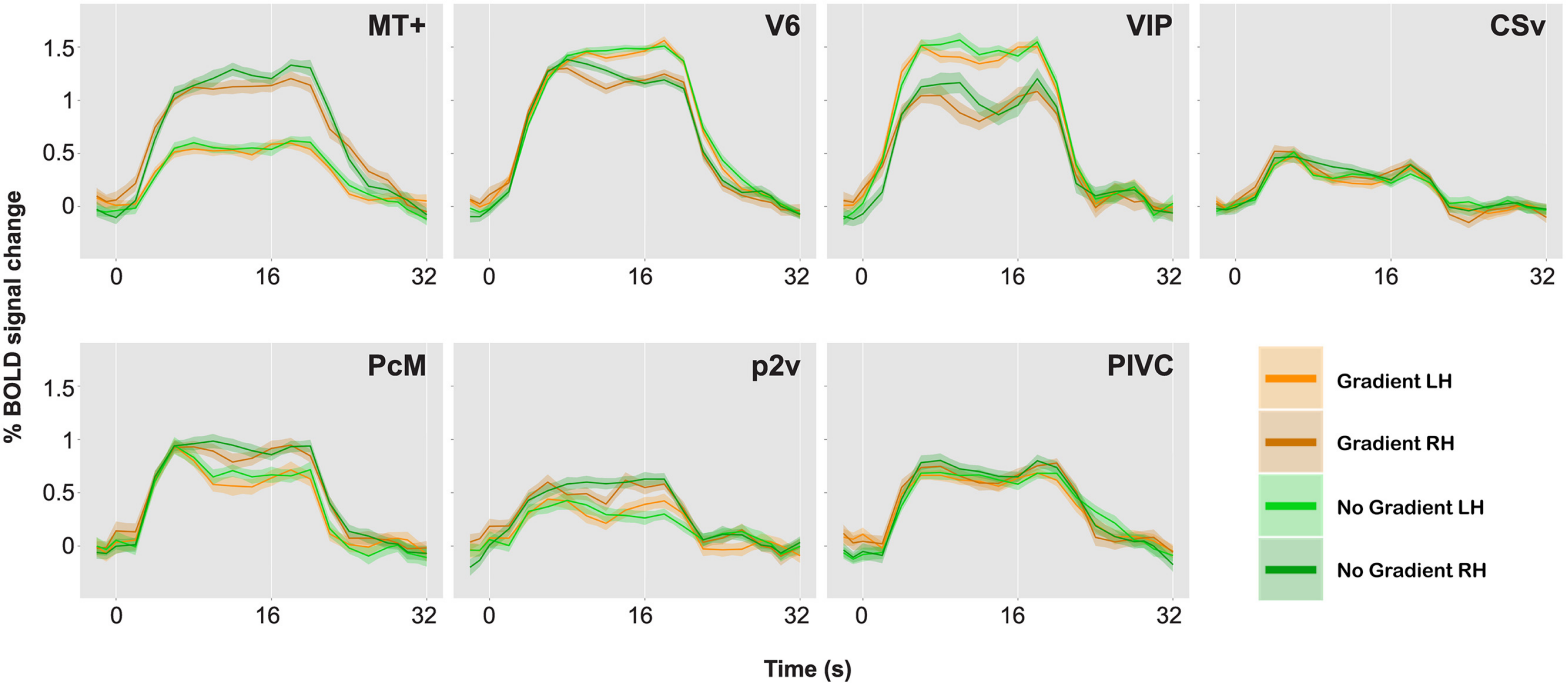
**BOLD responses to optic-flow stimuli with and without speed gradient, for MT+, V6, ventral intra-parietal area (VIP), cingulate sulcus visual area (CSv), precuneus motion area (PcM), p2v, and parieto insular vestibular cortex (PIVC) in one representative brain**. Time series data for the two conditions and for the two hemispheres are overlaid. A single time series was computed from 10 runs. The time series was then collapsed over a single stimulus cycle of 32 s: The stimulus-presentation block lasted from 0 to 16 s followed by a 16-s ISI. Error bars indicate SE.

**Figure 4** represents the percent signal changes in the gradient and the no-gradient conditions in one of the participants. All seven ROIs in all hemispheres showed positive BOLD responses to optic-flow stimulation in both the gradient and the no-gradient conditions, which confirms the sensitivity and selectivity of those areas to optic flow documented in the literature (Cardin and Smith, 2010, 2011). In order to examine whether any of ROIs showed differential responses to optic-flow stimuli with and without speed gradient, an event-related average time series was computed for each of the 10 experimental runs for each ROI, and magnitude of BOLD responses between 5 s after the stimulus onset and 4 s after the stimulus offset was averaged for both conditions. Consequently, there were 10 data points per condition per ROI. A paired two-sample *t*-test (two-tailed) was performed for each ROI in each participant. The results of the *t*-tests are reported in **Table 2**.

**Table 2:** Results of comparison of BOLD responses between gradient and no-gradient conditions.

Participant	Hemisphere	MT+	V6	VIP	CSv	PcM	P2v	PIVC
**HA**	LH	0.30 (0.77)	1.17 (0.27)	1.27 (0.24)	0.72 (0.49)	1.27 (0.24)	1.80 (0.11)	1.79 (0.11)
	RH	1.43 (0.19)	0.72 (0.49)	0.50 (0.70)	2.78 (0.02)*	3.49 (0.007)**	1.71 (0.12)	1.87 (0.09)
**JS**	LH	−1.54 (0.16)	−0.02 (0.98)	−0.40 (0.70)	−0.61 (0.56)	0.32 (0.76)	−1.95 (0.08)	0.17 (0.87)
	RH	−2.11 (0.06)	−0.13 (0.90)	0.01 (0.99)	−0.23 (0.82)	0.87 (0.41)	−1.13 (0.29)	−1.48 (0.17)
**BB**	LH	0.66 (0.53)	0.49 (0.63)	1.26 (0.24)	0.50 (0.63)	0.94 (0.37)	0.34 (0.74)	0.62 (0.54)
	RH	2.39 (0.04)*	0.76 (0.47)	1.20 (0.26)	0.17 (0.87)	1.05 (0.32)	1.97 (0.08)	0.83 (0.43)

Test statistics (*t*) and p-level (in parentheses) are reported for each ROI.

*p < 0.05, **p < 0.01.

As can be seen in **Table 2**, few of the ROIs exhibited differential activation between the gradient and the no-gradient conditions. The neural responses were similar in the two conditions in most/all of ROIs in all three participants (**Table 2**); the exceptions were areas CSv [*t*(9) = 2.78, *p* = 0.02] and PcM [*t*(9) = 3.49, *p* = 0.007] in the right hemisphere of one participant, and area MT+ [*t*(9) = 2.39, *p* = 0.04] in the right hemisphere of another. There was no consistency in areas that showed a significant difference between the two conditions across participants.

#### Effect of Vection

In order to assess the role of optic-flow selective areas in induction of vection, and therefore in the processing of optic flow as a cue to self-motion, activation during the vection events was measured against that during the no-vection events.

**FIGURE 5:**
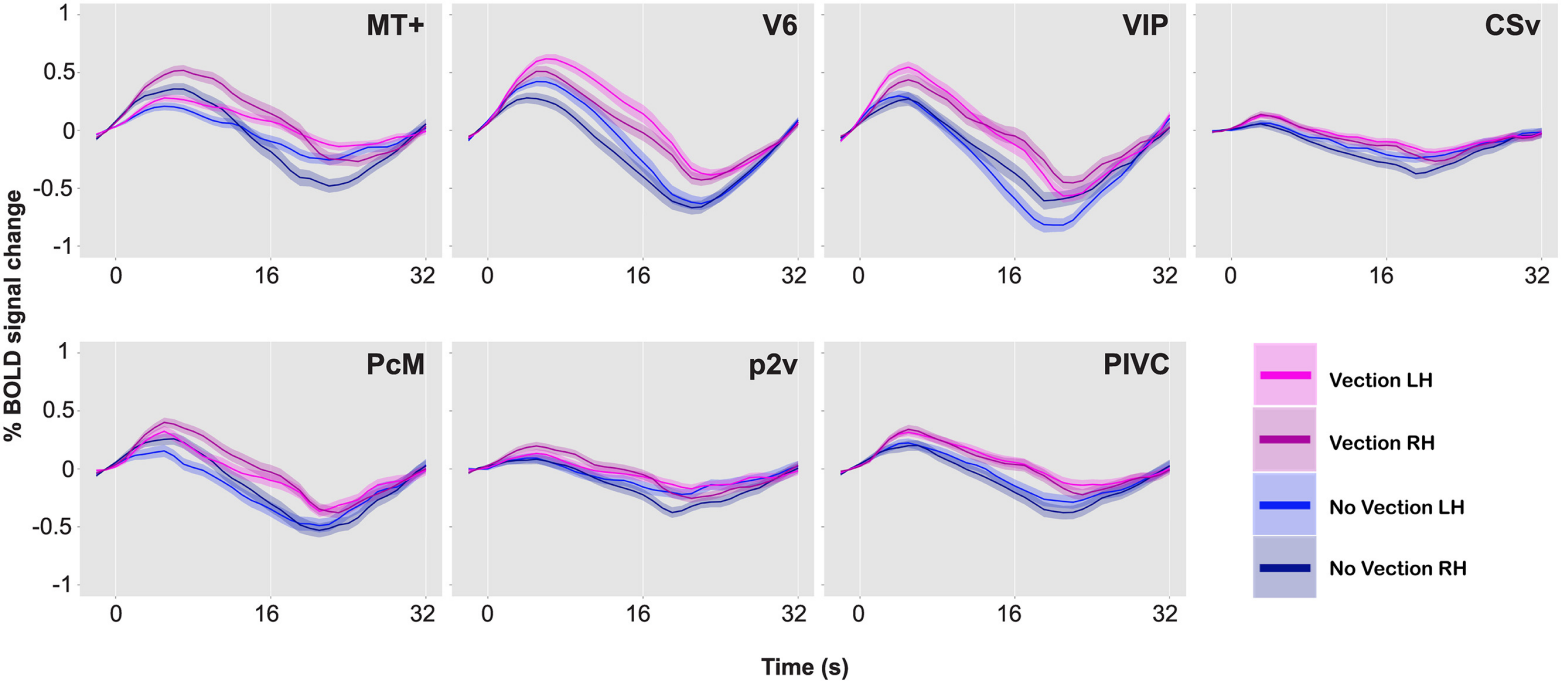
**BOLD responses during vection and no-vection events, for MT+, V6, VIP, CSv, PcM, p2v, and PIVC in one representative brain**. Time series data for the two types of events and for the two hemispheres are overlaid. A single time series was computed from 10 runs. The time series was then collapsed over a single cycle of 32 s. Error bars indicate SE.

**Figure 5** illustrates the percent signal changes during the vection and the no-vection events in one of the participants. A paired two-sample *t*-test (two-tailed) was performed for each ROI in each participant to compare activity in each area during the vection events to that during the no-vection events. For both sets of events, an event-related average time series was computed for each of the 10 experimental runs for each ROI, and magnitude of BOLD responses across 10 s from 5 s after the button press was averaged. Consequently, there were 10 data points per type of events per ROI. The results of the *t*-tests are reported in **Table 3**.

**Table 3:** Results of comparison of BOLD responses during self-reported states of vection and no-vection.

Participant	Hemisphere	MT+	V6	VIP	CSv	PcM	P2v	PIVC
**HA**	LH	2.88 (0.02)*	2.53 (0.03)*	1.98 (0.08)	2.30 (0.04)*	1.98 (0.07)	0.27 (0.79)	3.15 (0.01)*
	RH	3.77 (0.004)**	3.29 (0.009)**	3.53 (0.006)**	1.03 (0.33)	1.87 (0.09)	−0.58 (0.58)	2.37 (0.04)*
**JS**	LH	1.69 (0.13)	2.33 (0.04)*	1.97 (0.08)	1.48 (0.17)	1.77 (0.11)	0.59 (0.57)	−0.09 (0.93)
	RH	2.19 (0.06)	2.25 (0.05)	2.40 (0.04)*	1.68 (0.13)	2.06 (0.07)	1.11 (0.29)	2.89 (0.02)*
**BB**	LH	2.36 (0.04)*	4.02 (0.003)**	4.79 (0.001)**	3.27 (0.01)**	4.36 (0.002)**	2.61 (0.03)*	3.61 (0.006)**
	RH	3.55 (0.006)**	3.98 (0.003)**	3.20 (0.01)*	4.40 (0.002)**	3.85 (0.004)**	4.33 (0.002)**	4.93 (0.001)**

Test statistics (*t*) and p-level (in parentheses) are reported for each ROI.

*p < 0.05, **p < 0.01.

A comparison of BOLD responses between the vection and the no-vection events in each ROI yielded that magnitude of activation in areas including MT+, V6, VIP, and PIVC was significantly larger during periods of self-reported perception of vection (**Table 3**). Those four areas exhibited differential activation in more than four of the six hemispheres.

## Discussion

### Functional Localization of ROIs

Results confirm the effectiveness of the functional localizer employed in this study, which is based upon that proposed by Pitzalis et al. (2010), in localizing not only V6 but also other optic-flow selective regions (Cardin and Smith, 2010, 2011). It is interesting to note that, contrarily to what has been suggested in the literature (Pitzalis et al., 2006; Cardin et al., 2012), the results suggest that wide-field stimulation is not necessarily crucial for localization of V6 amongst other ROIs of this study.

### Speed Gradient vs. Perceived Vection

The comparison of activity in ROIs between the gradient and the no-gradient conditions yielded unremarkable results: There was not one area that consistently responded differently to optic-flow stimuli with and without a speed gradient across participants. Speed gradients are one of the defining factors of optic flow that makes it consistent with optic-flow stimulation that is experienced by an observer during actual self-motion, and this indeed modulated the duration of vection perceived by participants during the experiments. The lack of clear difference in BOLD responses between the gradient and the no-gradient conditions, however, suggests that these cortical sensory areas are not simply modulated by this physical property of optic flow.

The principal purpose of these experiments was to assess whether optic flow is encoded differently according to the presence or absence of vection. Results of the contrast between BOLD responses during the vection and the no-vection events show that there are several optic-flow selective regions that respond more robustly during self-reported perceptual states of vection. In addition to the vestibular area PIVC, these areas include visual areas MT+ and V6, and the multisensory area VIP.

### Visual Areas MT+ and V6

Because both MT+ and V6 have been shown to possess selectivity toward coherent optic-flow stimuli (Morrone et al., 2000; Dukelow et al., 2001; Huk et al., 2002; Wall and Smith, 2008; Cardin and Smith, 2010, 2011; Pitzalis et al., 2010), they might be expected to show consistent activation regardless of whether vection is experienced. However, in line with previous findings (Kovacs et al., 2008; Wall and Smith, 2008; Cardin and Smith, 2010), differential activation was observed in visual areas MT+ and V6 depending on the presence or absence of vection.

The most plausible explanation for these results may be that those visual areas receive feedback from the multisensory areas (e.g., VIP) that receive direct input from PIVC (Lewis and van Essen, 2000). Although evidence in the human brain is limited, there are findings in the monkey brain that indicate that MT+ is anatomically and functionally connected to the multisensory areas VIP (Maunsell and van Essen, 1983; Ungerleider and Desimone, 1986; Boussaoud et al., 1990; Baizer et al., 1991) and the precuneus (Blum et al., 1950; Leichnetz, 2001). Furthermore, MT+ and V6 in the human brain have recently been found to be anatomically connected via ventral occipital fasciculus (VOF: Yeatman et al., 2014). This reciprocal relationship between the visual and multisensory areas would allow for optic flow as a cue to self-motion to be processed more efficiently.

### Multisensory Area VIP

Results also indicate that the multisensory area VIP respond differently to optic flow during the periods in which vection is perceived. This area is considered a part of the dorsal visual pathway in the monkey brain (Felleman and van Essen, 1991); and has been shown to consist of a substantial number of multisensory neurons, and to respond to all visual, auditory, and somatosensory stimulation (Duhamel et al., 1998; Bremmer et al., 2001a,b). It has also been suggested that VIP receive visual projection from MT+ (Maunsell and van Essen, 1983; Ungerleider and Desimone, 1986) as well as vestibular projection from PIVC (Lewis and van Essen, 2000) in the monkey brain. Those findings and the results of this study place the multisensory area VIP in a good position to integrate visual cues related to self-motion and vestibular information (or lack thereof). The possibility that VIP integrates sensory information and is an extremely important area for processing self-motion is further supported by the findings that VIP interacts with premotor area (Schlack et al., 2002; Klam and Graf, 2003) of which function is to execute motion that is driven by sensory information.

### Vestibular Area PIVC

The vestibular area PIVC (Guldin and Grusser, 1998) showed differential activation during states of vection, which suggests that PIVC encodes optic flow differently depending on the presence or absence of vection. This agrees with previous findings (Brandt, 1999; Kleinschmidt et al., 2002; Deutschlander et al., 2004; Indovina et al., 2005; Kovacs et al., 2008), and is consistent with the finding that PIVC is connected with VIP (Lewis and van Essen, 2000), which also showed the same pattern of differential activation.

It should be noted, however, in contrast to the findings of a number of previous studies (Brandt et al., 1998; Brandt and Dieterich, 1999; Kleinschmidt et al., 2002; Deutschlander et al., 2004), increased rather than decreased activity was observed in PIVC. This discrepancy in the findings could be due to the motion components that constituted the optic-flow stimuli used in this study. Unlike in some of the previous studies, which used constant-velocity stimuli with single motion component, the stimuli used in this study consisted both changing linear-motion (i.e., expansion and contraction) and rotational-motion components in order to maximize BOLD responses in the ROIs. It has been shown that PIVC responds selectively to body acceleration (Nishiike et al., 2002; Indovina et al., 2005); therefore it is likely that using stimuli with a dynamic pattern of motion compatible with continuously changing body acceleration (in forward, backward, and rotating directions) lead to robust and positive BOLD responses in PIVC as well as stronger visuo-vestibular interaction observed in this study.

### Individual Differences

Kennedy et al. (1996) demonstrated that there are vast individual differences in magnitude of vection experienced and the duration for which perception of vection lasts.

One participant who was extremely susceptible to vection coincidentally exhibited the largest and most persistent differential activation in the areas discussed above according to the presence or absence of vection. It is possible that the individual differences in brain activity reflect those observed at the behavioral level; however, it is difficult to draw any inference from the results of this study alone. In order to address this question, not only the duration of perceived vection but also the magnitude of vection should be taken into account and it is critical that those measures are directly correlated with brain activity.

### Futher Directions

The findings not only dissociate the roles of and interaction between the cortical sensory ROI related to optic-flow stimulation from those related to sensation of self-motion, but also contribute to discussion on multisensory integration processes underlying perception of vection. Future studies should aim to validate and strengthen evidence for this multisensory mechanism, possibly by introducing analyses such as multi-voxel pattern analysis (MVPA). Inclusion of MVPA could shed new light to the interpretation of the data by increasing sensitivity to changes in cortical activation between the two perceptual states of vection and no vection (Arnoldussen et al., 2013).

## Conclusion

The present study investigated how vection is represented in the optic-flow selective sensory areas to determine which of those areas are involved in the processing of optic flow as a visual cue to self-motion. It was found that activation in visual areas MT+, V6, multisensory area VIP, and vestibular area PIVC seems to reflect vection, and that VIP is the most likely candidate for an area that integrates visual information related to self-motion and vestibular information.

## Conflict of Interest Statement

The authors declare that the research was conducted in the absence of any commercial or financial relationships that could be construed as a potential conflict of interest.

## References

[B1] Arnoldussen D. M. Goossens J. van den Berg A. V. (2013). Differential responses in dorsal visual cortex to motion and disparity depth cues. *Front. Hum. Neurosci.* 7:815 10.3389/fnhum.2013.00815 PMC3857528 24339808

[B2] Baizer J. S. Ungerleider L. G. Desimone R. (1991). Organization of visual inputs to the inferior temporal and posterior parietal cortex in macaques. *J. Neurosci.* 11 168–190. 1702462 10.1523/JNEUROSCI.11-01-00168.1991 PMC6575184

[B3] Blum J. S. Chow K. L. Pribram K. (1950). A behavioral analysis of the organisation of the parieo-temporo-preoccipital cortex. *J. Comp. Neurol.* 93 55–100. 10.1002/cne.900930105 14778908

[B4] Boussaoud D. Ungerleider L. G. Desimone R. (1990). Pathways for motion analysis: cortical connections of the medial superior temporal and fundus of the superior temporal visual areas in the macaque. *J. Comp. Neurol.* 296 462–495. 10.1002/cne.902960311 2358548

[B5] Brandt T. (1999). Cortical visual-vestibular interaction for spatial orientation and self-motion perception. *Curr. Opin. Neurol.* 12 1–4. 10.1097/00019052-199902000-00001 10097877

[B6] Brandt T. Bartenstein P. Janek A. Dieterich M. (1998). Reciprocal inhibitory visual-vestibular interaction – visual motion stimulation deactivates the parieto-insular vestibular cortex. *Brain* 121 1749–1758. 10.1093/brain/121.9.1749 9762962

[B7] Brandt T. Dieterich M. (1999). The vestibular cortex: its locations, functions and disorders. *Ann. N. Y. Acad. Sci.* 871 293–312. 10.1111/j.1749-6632.1999.tb09193.x 10372080

[B8] Bremmer F. Schlack A. Ahah N. Zafiris O. Kubischik M. Hoffmann K.-P. (2001a). Polymodal motion processing in posterior parietal and premotor cortex: a human fMRI study strongly implies equivalencies between humans and monkeys. *Neuron* 23 287–296. 10.1016/S0896-6273(01)00198-2 11182099

[B9] Bremmer F. Schlack A. Fuhamel J. R. Graf W. Fink G. R. (2001b). Space coding in primate posterior parietal cortex. *Neuroimage* 14 S46–S51. 10.1006/nimg.2001.0817 11373132

[B10] Cardin V. Sherrington R. Hemsworth L. Smith A. T. (2012). Human V6: Functional characterisation and localisation. *PLoS ONE* 7:e47685 10.1371/journal.pone.0047685 PMC3480433 23112833

[B11] Cardin V. Smith A. T. (2010). Sensitivity to human visual and vestibular cortical regions to egomotion-compatible visual stimulation. *Cereb. Cortex* 20 1964–1973. 10.1093/cercor/bhp268 20034998 PMC2901022

[B12] Cardin V. Smith A. T. (2011). Sensitivity of human visual cortical area V6 to stereoscopic depth gradients associated with self-motion. *J. Neurophysiol.* 106 1240–1249. 10.1152/jn.01120.2010 21653717 PMC3174812

[B13] Deutschlander A. Bense S. Stephan T. Schwaiger M. Dieterich M. Brandt T. (2004). Rollvection versus linearvection: comparison of brain activations in PET. *Hum. Brain Mapp.* 21 143–153. 10.1002/hbm.10155 14755834 PMC6871853

[B14] Dichgans J. Brandt T. (1978). “Visual-vestibular interaction: effects on self-motion perception and postual control,” in *Handbook of Sensory Physiology*, Vol. 8 eds Held R. Leibowitz H. W. Teuber H.-L. (Berlin: Springer), 755–804.

[B15] Duffy D. J. Wurtz R. H. (1991a). Sensitivity to MST neurons to optic flow stimuli. II. Mechanisms of response selectivity revealed by small-field stimuli. *J. Neurophysiol.* 65 1346–1359. 1875244 10.1152/jn.1991.65.6.1346

[B16] Duffy D. J. Wurtz R. H. (1991b). Sensitivity of MST neurons to optic flow stimuli. I. A contimuum of response selectivity to large-field stimuli. *J. Neurophysiol.* 65 1329–1345. 1875243 10.1152/jn.1991.65.6.1329

[B17] Duffy D. J. Wurtz R. H. (1995). Responses of monkey MST neuron to optic flow stimuli with shifted centres of motion. *J. Neurosci.* 15 5192–5208. 7623145 10.1523/JNEUROSCI.15-07-05192.1995 PMC6577859

[B18] Duhamel J. R. Colby C. L. Goldberg M. E. (1998). Ventral intraparietal area of the macaque: visual and somatic response properties. *J. Neurophysiol.* 79 126–136. 9425183 10.1152/jn.1998.79.1.126

[B19] Dukelow S. P. DeSouza J. F. Culham J. C. van den Berg A. V. Menon R. S. Vilis T. (2001). Distinguishing subregions of the human MT+ complex using visual fields and pursuit eye movements. *J. Neurophysiol.* 86 1991–2000. 11600656 10.1152/jn.2001.86.4.1991

[B20] Felleman D. J. van Essen D. C. (1991). Distributed hierarchical processing in the primate cerebral cortex. *Cereb. Cortex* 1 1–47. 10.1093/cercor/1.1.1 1822724

[B21] Gibson J. J. (1950). *The Perception of the Visual World.* Boston: Houghton Miﬄin.

[B22] Gibson J. J. (1954). The visual perception of objective motion and subjective movement. *Psychol. Rev.* 61 304–314. 10.1037/h0061885 13204493

[B23] Guldin W. O. Grusser O. J. (1998). Is there a vestibular cortex? *Trends Neruosci.* 21 254–259. 10.1016/S0166-2236(97)01211-3 9641538

[B24] Howard I. P. Templeton W. B. (1966). *Human Spatial Orientation.* Oxford: John Wiley & Sons.

[B25] Huk A. C. Dougherty R. F. Heeger D. J. (2002). Retinotopy and functional subdivision of human areas MT and MST. *J. Neurosci.* 22 7195–7205. 12177214 10.1523/JNEUROSCI.22-16-07195.2002 PMC6757870

[B26] Indovina I. Maffei V. Bosco G. Zago M. Macaluso E. Lacquaniti F. (2005). Representation of visual gravitational motion in the human vestibular cortex. *Science* 308 416–419. 10.1126/science.1107961 15831760

[B27] Kennedy R. S. Hettinger L. J. Harm D. L. Ordy J. M. Dunlap W. P. (1996). Psychological scaling of circular vection (CV) produced by optokinetic (OKN) motion: individual differences and effects of practice. *J. Vestib. Res.* 6 331–341. 10.1016/0957-4271(96)00002-X 8887891

[B28] Klam F. Graf W. (2003). Vestibular response kinematics in posterior parietal cortex neurons of macaque monkeys. *Eur. J. Neurosci.* 18 995–1010. 10.1046/j.1460-9568.2003.02813.x 12925025

[B29] Kleinschmidt A. Thilo K. V. Buchel C. Gresty M. A. Bronstein A. M. Frackwiak R. S. (2002). Neural correlates of visual-motion perception as object- or self-motion. *Neuroimage* 16 873–882. 10.1006/nimg.2002.1181 12202076

[B30] Koenderink J. J. (1986). Optic flow. *Vision Res.* 26 161–179. 10.1016/0042-6989(86)90078-7 3716209

[B31] Kovacs G. Raabe M. Greenlee M. W. (2008). Neural correlates of visually induced self-motion illusion in depth. *Cereb. Cortex* 18 1779–1787. 10.1093/cercor/bhm203 18063566

[B32] Lappe M. Bremmer F. van den berg A. V. (1999). Perception of self-motion from visual flow. *Trends Cogn. Sci.* 3 329–226. 10.1016/S1364-6613(99)01364-9 10461195

[B33] Leichnetz G. R. (2001). Connections of the medial posterior parietal cortex (area 7m) in the monkey. *Anat. Rec.* 263 215–236. 10.1002/ar.1082 11360237

[B34] Lewis J. W. van Essen D. C. (2000). Corticocortical connections of visual sensorimotor and multimodal processing areas in the parietal lobe of the macaque monkey. *J. Comp. Neurol.* 428 112–137. 10.1002/1096-9861(20001204)428:1<112::AID-CNE8>3.0.CO;2-9 11058227

[B35] Maunsell J. H. van Essen D. C. (1983). Functional properties of neurons in the middle temporal visual area of the macaque monkey. I. Selectivity for stimulus direction, speed, and orientation. *J. Neurophysiol.* 49 1127–1147. 6864242 10.1152/jn.1983.49.5.1127

[B36] Morrone M. C. Tosetti M. Montanaro D. Fiorentini A. Cioni G. Burr D. C. (2000). A cortical area that responds specifically to optic flow revealed by fMRI. *Nat. Neurosci.* 3 1322–1328. 10.1038/81860 11100154

[B37] Nishiike S. Nakagawa S. Nakagawa A. Uno A. Tonoike M. Takeda N. (2002). Magnetic cortical responses evoked by visual linear forward acceleration. *Neuroreport* 13 1805–1808. 10.1097/00001756-200210070-00023 12395128

[B38] Pitzalis S. Galletti C. Huang R.-S. Patria F. Committeri G. Galati G. (2006). Wide-field retinotopy defines human cortical visual area v6. *J. Neurosci.* 26 7962–7973. 10.1523/JNEUROSCI.0178-06.2006 16870741 PMC6674231

[B39] Pitzalis S. Sereno M. I. Committeri G. Fattori P. Galati G. Patria F. (2010). Human V6: the medial motion area. *Cereb. Cortex* 20 41–424. 10.1093/cercor/bhp112 PMC2803738 19502476

[B40] Schlack A. Hoffmann K. P. Bremmer F. (2002). Interaction of linear vestibular and visual stimulation in the macaque ventral intraparietal area (VIP). *Eur. J. Neurosci.* 16 1877–1886. 10.1046/j.1460-9568.2002.02251.x 12453051

[B41] Ungerleider L. G. Desimone R. (1986). Cortical connections of visual area MT in the macaque. *J. Comp. Neurol.* 248 190–222. 10.1002/cne.902480204 3722458

[B42] Wall M. Smith A. T. (2008). The representation of egomotion in the human brain. *Curr. Biol.* 18 191–194. 10.1016/j.cub.2007.12.053 18221876

[B43] Warren W. H. Hannon D. J. (1988). Direction of self-motion is perceived from optical flow. *Nature* 336 162–163. 10.1038/336162a0

[B44] Wertheim A. (1994). Motion perception during self-motion – the direct versus inferential controversy revisited. *Behav. Brain Sci.* 17 293–311. 10.1017/S0140525X00034646

[B45] Wexler M. Panerai F. Lamouret I. Droulez J. (2001). Self-motion and the perception of stationary objects. *Nature* 409 85–88. 10.1038/35051081 11343118

[B46] Yeatman J. D. Weiner K. S. Pestilli F. Rokem A. Mezer A. Wandell B. A. (2014). The vertical occipital fasciculus: A century of controversy resolved by in vivo measurements. *Proc. Natl. Acad. Sci. U.S.A.* 111 E5214–E5223. 10.1073/pnas.1418503111 25404310 PMC4260539

